# Sodium to globulin ratio as a prognostic factor for patients with advanced gastric cancer

**DOI:** 10.7150/jca.47314

**Published:** 2020-10-23

**Authors:** Liqun Zhang, Zhuo Wang, Jiawen Xiao, Hao Chen, Zhiyan Zhang, Haijing Li, Yuanhe Wang, Haiyan Piao, Fang Li, Lisha Zhang, Jingdong Zhang

**Affiliations:** 1 Medical Oncology Department of Gastrointestinal Cancer, Liaoning Cancer Hospital & Institute, Cancer Hospital of China Medical University, No.44 Xiaoheyan Road, Dadong District, Shenyang 110042, Liaoning Province, China.; 2 Department of Medical Oncology, Shenyang Fifth People Hospital, Tiexi District, Shenyang 110020, Liaoning Province, China.; 3 Department of Medical Oncology, Liaohua Hospital, Hongwei District, Liaoyang 111003, Liaoning Province, China.; 4 Department of Hepatobiliary Surgery, Liaoning Cancer Hospital & Institute, Cancer Hospital of China Medical University, No.44 Xiaoheyan Road, Dadong District, Shenyang 110042, Liaoning Province, China.; 5 Department of Gastrointestinal Surgery, The Second Hospital Affiliated to Harbin Medical University, No. 246, Xuefu Road, Nangang District, Harbin 150086, Heilongjiang Province, China.

**Keywords:** sodium to globulin ratio, gastric cancer, first-line chemotherapy, prognosis, progression-free survival, overall survival

## Abstract

**Background:** Electrolyte disturbance and systemic inflammation contributes to poor prognosis of cancer patients. Levels of serum sodium and globulin can reflect electrolyte homeostasis and inflammatory state, respectively, therefore have potential as prognostic factors for cancer patients. In this study, we hypothesized that sodium to globulin ratio (SGR) could have superior accuracy in predicting cancer patient survival, than sodium and globulin alone. We therefore sought to investigate its efficacy in prognosis of patients with advanced gastric cancer (GC) receiving first-line chemotherapy.

**Methods:** A total of 265 patients, with advanced GC, were recruited in this retrospective study from January 2014 to January 2019. We first determined SGR cut-off values using the receiver operating characteristic (ROC) analysis, then analyzed the relationship between pretreatment SGR and clinicopathological features and the effect of chemotherapy. Finally, we evaluated progression-free survival (PFS) and overall survival (OS) rates of the entire and subgroup populations using univariate and multivariate logistic regressions.

**Results:** SGR recorded a cut-off value of 5.54, and had a significantly higher area under the curve (AUC) value (0.619, *p* = 0.001) than fibrinogen (0.575, *p* = 0.034) and albumin (0.610, *p* = 0.002) alone. Organ metastasis, and peritoneal invasion ratios, as well as neutrophil and CA72-4 levels varied significantly between the low-SGR (SGR≤ 5.54) and high SGR (SGR> 5.54) groups (all *p* < 0.05). Specifically, patients in the low-SGR group exhibited significantly lower disease control rates (83.4%) than those in the high-SGR group (97.2%) (*p* < 0.001). Results from multivariate analysis indicated that high-SGR was an independent risk factor for PFS (Hazard ratio [HR]: 0.539, p < 0.001) and OS (HR: 0.574, p < 0.001). Moreover, patients in the low-SGR group exhibited significantly worse PFS (134 vs. 221 days, *p* < 0.001) and OS (311 vs. 420 days, *p* < 0.001) than those in the high-SGR group. Furthermore, subgroup analysis revealed that SGR was still a powerful prognostic indicator in GC patients with good prognosis or normal biochemical indexes, including no peritoneal infiltration, normal neutrophil counts, and normal serum sodium and globulin levels (all *p* < 0.001).

**Conclusions:** Overall, our findings indicate that SGR is a novel and promising prognostic factor for GC patients. It has superior accuracy, to sodium and globulin alone, hence it is a powerful tool for evaluating effects of treatment, PFS, and OS in patients with advanced GC, who receive first-line chemotherapy.

## Introduction

Gastric cancer (GC), one of the most common malignancies, has been associated with high mortality rates 1. Technological advancement in gastric cancer screening has significantly improved detection of early GC patients, resulting in a high (90%) five-year survival rate of early-stage GC patients, following curative treatment. However, for many patients with advanced stage GC, lack of or mild cancer symptoms at diagnosis, implies they cannot undergo curative resection. Despite numerous research efforts being directed towards development of various therapeutic strategies, the median survival rates for patients with advanced-stage GC remains at only 13 months^2^. To improve survival rates in this group of patients, it is imperative to identify novel and effective prognostic factors.

Previous studies have implicated various pathophysiological factors, such as electrolyte disturbance and systemic inflammation in poor outcomes of cancer patients 3-5. In addition, serum sodium and globulin concentrations have been employed as biochemical indicators for routine laboratory tests. In fact, previous studies have shown that these indicators alone can objectively reflect electrolyte disturbance and inflammatory status of patients, thereby effectively predicting therapeutic response and prognosis of various tumors 6-9. On the other hand, the ratio of sodium to globulin (SGR) may result in superior efficacy in predicting prognosis of cancer patients, than sodium and globulin alone. However, only a handful of studies have reported the use of SGR as a prognostic factor in cancer.

In this study, we evaluated the effect of SGR before first-line chemotherapy on treatment response, progression-free survival (PFS), and overall survival (OS) of patients with advanced GC.

## Materials and Methods

### Patients

A total of 265 advanced GC patients, who received first-line chemotherapy upon admission to the Cancer Hospital of China Medical University, were enrolled in the study between January 2014 to January 2019. Patients were only included if they met the following criteria: (1) gastric cancer was confirmed pathologically; (2) metastasis was confirmed in organs using computed tomography (CT), magnetic resonance imaging (MRI) scans, or other imaging methods; (3) patients' TNM staging indicated that all cases were stage III-IV; (4) subjects completed no less than two cycles of first-line chemotherapy after the diagnosis of the disease; (5) patients had no acute infection and received nutritional support; and (6) a majority of the patients were treated with a first-line cytotoxic drug: oxaliplatin, cisplatin, fluorouracil, and docetaxel. Ethical approval was obtained from the Cancer Hospital of China Medical University Ethics Committee, prior to conducting the study.

### Definitions

A blood sample was collected from each patient, one week prior to cancer treatment. Standard reference ranges for serum sodium, globulin, potassium, albumin, blood urea nitrogen (BUN), creatinine (CREA), hemoglobin, neutrophil counts, thrombocyte counts, carcinoembryonic antigen (CEA), carbohydrate antigen 19-9 (CA19-9) and carbohydrate antigen 72-4 (CA72-4) were 135-145 mmol/L, 20-35 g/L, 3.5-5.3 mmol/L, 35-55 g/L, 3.2-6.0 mmol/L, 59-105 µmoI/L, 115-155 g/L, 1.8-6.3×10^9^/L, 100-300×10^9^/L, 0-5 ng/ml, 0-37 U/ml, and 0-6 U/ml, respectively. In this study, we define the ratio of sodium (mmol/L) to globulin (g/L) as SGR.

We used the Response Evaluation Criteria in Solid Tumors (RECIST 1.1) to estimate the chemotherapy response, every 2-3 cycles, and followed the patients until death or the end of the follow-up period; January 2020. The primary study endpoints were PFS and OS, whereas secondary endpoint was the disease control rate (DCR). PFS was defined as the interval between first-line chemotherapy and disease progression or the last follow-up time without progression, whereas OS was defined as the time between date of first-line chemotherapy and mortality or the end of follow-up. On the other hand, DCR was defined as the rate of complete response (CR), partial response (PR), and stable disease (SD).

### Statistical analyses

Statistical analyses were performed using GraphPad Prism (GraphPad Software, www.graphpad.com) and SPSS (SPSS Inc., Chicago, IL, USA) software. We used the receiver operating characteristic (ROC) curve to calculate the cut-off values for SGR, sodium, and globulin. Additionally, we performed chi-square and Mann-Whitney U-tests to determine statistically significant differences between low- and high-SGR groups. Analysis of OS and PFS was performed using univariate and multivariate analyses. Furthermore, the Kaplan-Meier method and Log-rank test were used to plot survival curves and for comparisons, respectively. Data followed by *p*-value less than 0.05 were considered statistically significant.

## Results

### Optimal cut-off values and patient stratification

**Figure 1:**
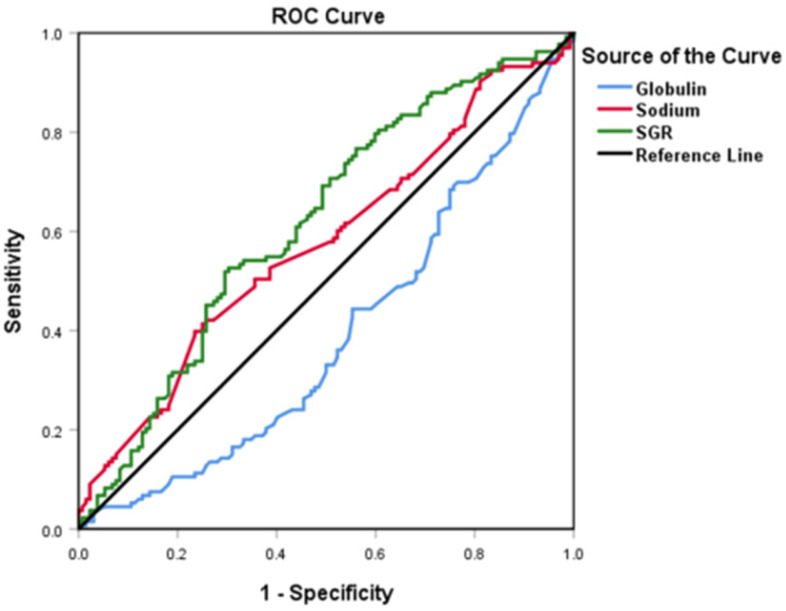
Profiles of globulin, sodium, and sodium to globulin ratio (SGR) in patients with advanced gastric cancer using ROC curves.

We used ROC curves to determine the optimal cut-off values for SGR, sodium, and globulin, (test) and OS (state) variables. Summarily, SGR, sodium, and globulin had optimal values of 5.54, 142.92, and 28.65, respectively. Moreover, we found AUC values of 0.619 (*p* = 0.001), 0.575 (*p* = 0.034) and 0.610 (*p* = 0.002) for SGR, sodium and globulin, respectively. The higher AUC in SGR indicated better prognostic accuracy in this variable, relative to the others. Consequently, participants with SGR > 5.54 were placed into the high-group, whereas those with a lower value stratified into the low-group (**Figure 1**).

### Correlation between clinicopathological features and SGR

**Figure 2:**
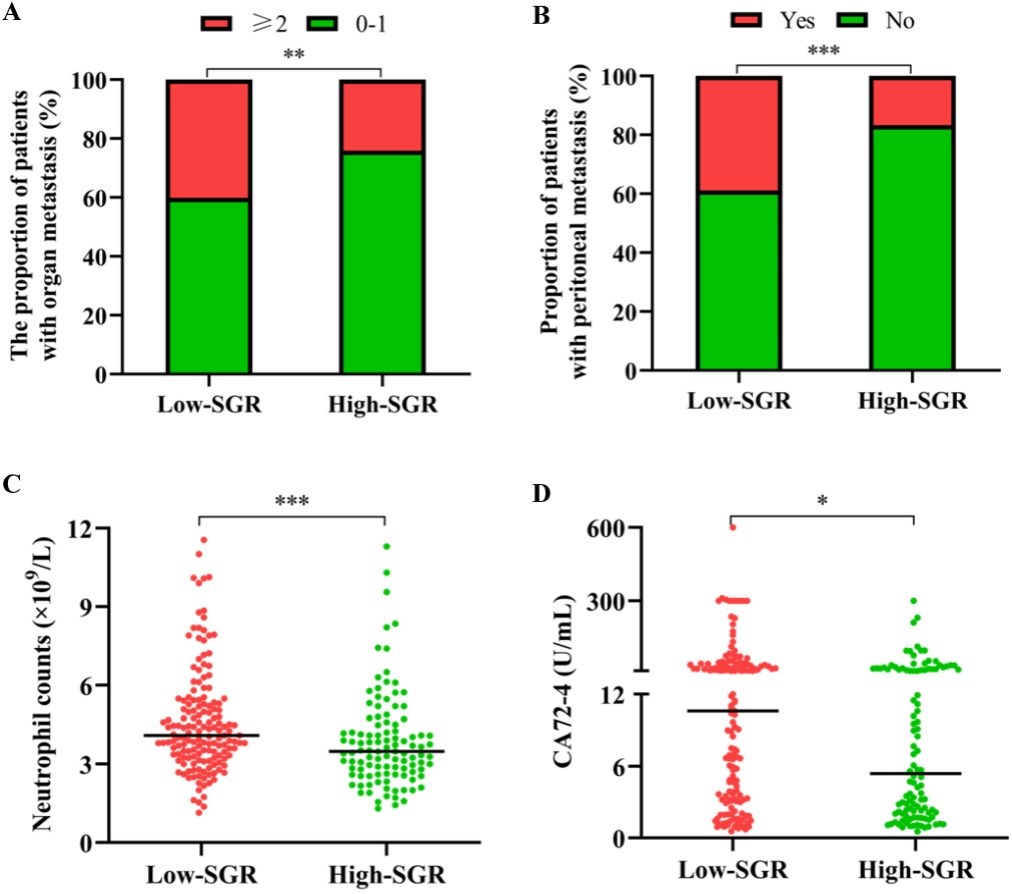
Relationship between the pretreatment sodium to globulin ratio (SGR) and (**A**) the number of metastatic organs, (**B**) peritoneal metastasis, (**C**) neutrophil count, and (**D**) carbohydrate antigen (CA) 72-4 level. * p < 0.05, ** *p* < 0.01, *** p < 0.001.

The median age of participants in our study cohort was 60 years, with 66.0% of all enrolled patients male. At the time of GC diagnosis, 33.6% of the patients had manifested metastases in more than one organ, 29.8% had developed peritoneal carcinomatosis, whereas 77.4% were found with stage IV gastric cancer. Analysis of the relationship between SGR levels and different patients' clinicopathological features revealed that those with low-SGR were more likely to have metastasis in no less than 2 organs (*p* = 0.007), peritoneal invasion (*p* < 0.001), higher neutrophil levels (*p* < 0.001) and CA72-4 (*p* = 0.014) (**Table 1 and Figure 2**).

### Relationship between SGR and response to first-line chemotherapy

**Figure 3:**
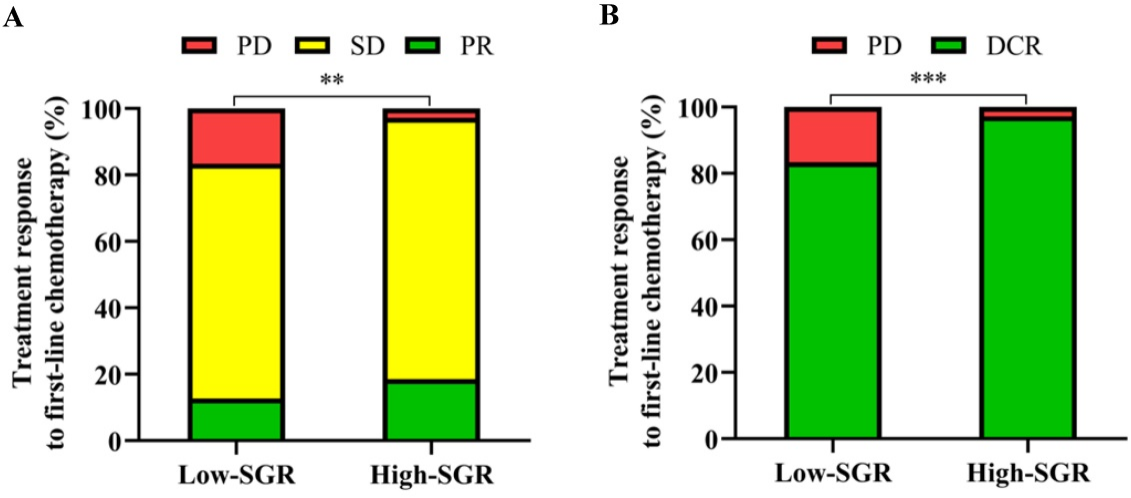
Relationship between pretreatment sodium to globulin ratio (SGR) and (**A**) progressive disease (PD), stable disease (SD), partial response (PR) and (**B**) disease control rate (DCR). ** *p* < 0.01, *** *p* < 0.001.

We found no patients with CR after first-line chemotherapy. However, patients in the high-SGR group exhibited higher proportions of SD and PR and lower proportions of PD (*p* = 0.001) relative to those in the low-SGR group. During first-line treatment, DCR rates of 97.2 and 83.4% (*p* < 0.001) were recorded in the high- and low-SGR groups, respectively (**Figure 3**).

### Prognostic factors predict patient survival

Results from univariate analyses revealed that SGR (*p* < 0.001), number of metastatic organs (*p* = 0.004), peritoneal metastasis (*p* < 0.001), CA72-4 (*p* = 0.006), and neutrophil counts (*p* = 0.012) were prognostic factors for PFS in the studied patients. On the other hand, multivariate analysis showed that SGR > 5.54 (Hazard ratio [HR]: 0.539, *p* < 0.001), no peritoneal metastasis (HR: 0.701, *p* = 0.031), and normal neutrophil counts (HR: 0.673, *p* = 0.042) had independent positive effects on PFS (**Table 2**). With regards to OS, results from univariate analyses indicated that SGR (*p* < 0.001), histological type (*p* = 0.016), number of metastatic organs (*p* = 0.012), peritoneal metastasis (*p* < 0.001), and neutrophil counts (*p* = 0.042) were prognostic factors. On the other hand, multivariate analyses revealed that SGR > 5.54 (HR: 0.574, *p* < 0.001), no peritoneal metastasis (HR: 0.655, *p* = 0.008) were independent prognostic factors for superior OS (**Table 3**).

**Figure 4:**
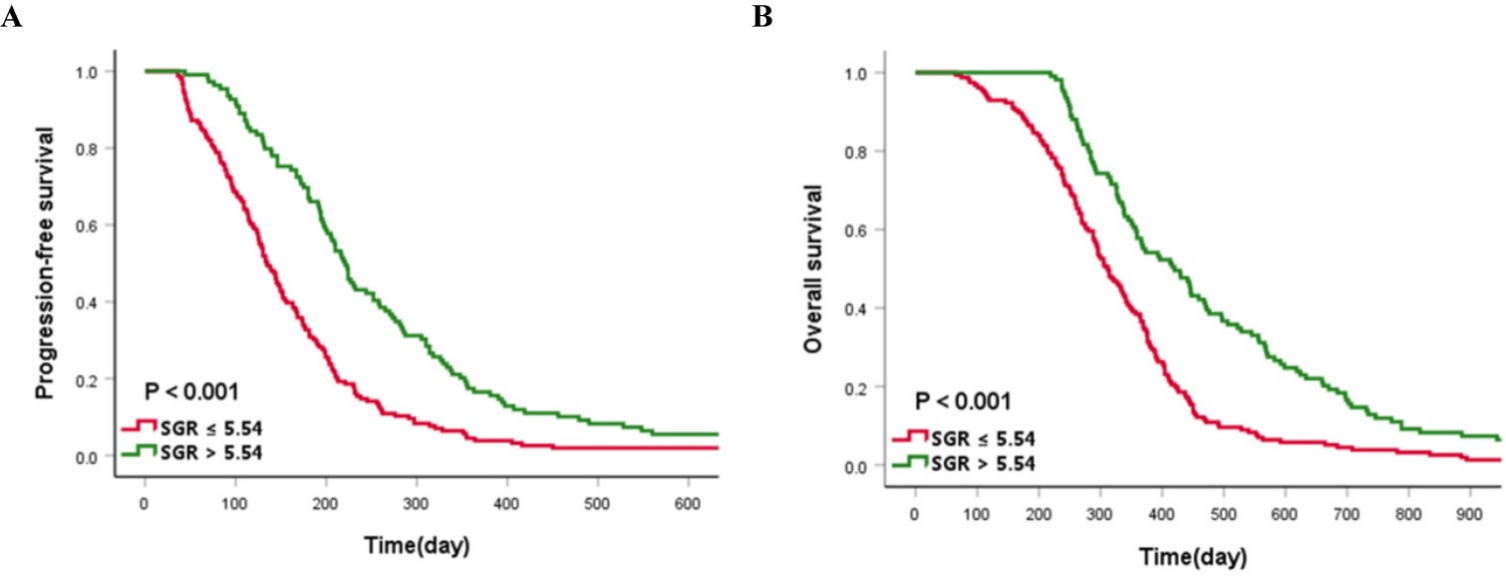
Kaplan-Meier curves of (**A**) progression-free survival and (**B**) overall survival according to the cut-off value of sodium to globulin ratio (SGR).

The median PFS and OS of the cases enrolled in this study were 173 and 345 days, respectively. Patients in the high-SGR group had a significantly longer median PFS (221 vs. 134 days, *p* < 0.001) and OS (420 vs. 311 days, *p* < 0.001) than those in the low-SGR group, respectively (**Figure 4**).

### Subgroup analysis

**Figure 5:**
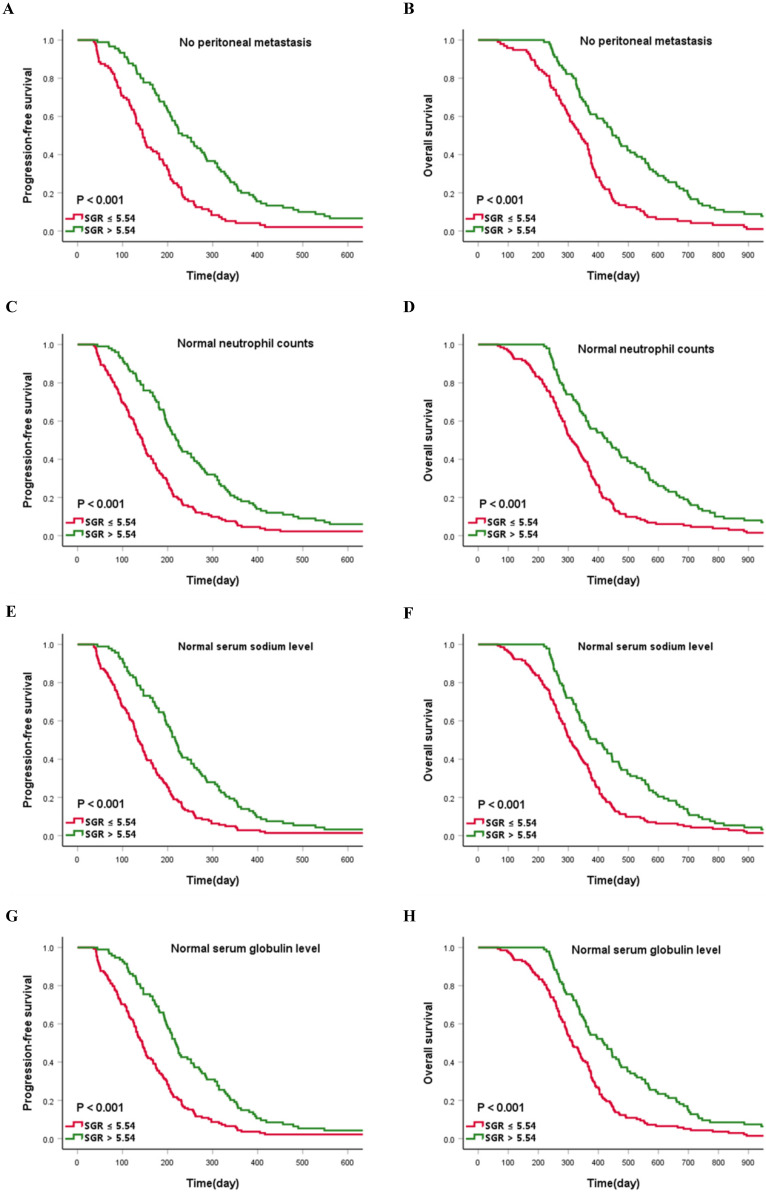
Kaplan-Meier curves describing survival for the cut-off value of sodium to globulin ratio (SGR) stratified by (**A and B**) no peritoneal metastasis, (**C and D**) normal neutrophil counts, (**E and F**) normal serum sodium level, and (**G and H**) normal serum globulin level.

Preliminary analysis indicated that no peritoneal infiltration and normal neutrophil counts (≤ 6.3×10^9^/L) were a protection factor for patient survival. Consequently, we assessed whether SGR validates prognosis determined by no peritoneal infiltration and normal neutrophil counts. Interestingly, patients in the high-SGR group, across no peritoneal metastasis and normal neutrophil count subgroups, still have prolonged PFS and OS relative to those in the low-SGR group (all *p* < 0.001). We hypothesized that clinicians may inadvertently ignore potentially critical patients with normal biochemical indexes. Consequently, we investigated whether SGR is a reliable prognostic indicator for patients with normal serum sodium (135-145 mmol/L) and globulin (20-35 g/L) levels. Summarily, patients in the high-SGR group, with normal serum sodium and globulin levels, still exhibited favorable prognosis compared with those in the low-SGR group (all *p* < 0.001) (**Figure 5**).

## Discussion

Numerous research efforts on prognostic cancer markers have generated a series of indicators for evaluating survival of patients with different tumors, such as gastric cancer 10-13. Serum sodium and globulin concentration alone are cheap and readily available routine blood examination strategies that effectively reflect outcomes and survival rates of patients with diverse malignancies, including gastric cancer 8, 9, 14-17. A combination of sodium and globulin, commonly termed sodium to globulin ratio, exhibits superior prognostic accuracy and precision relative to that of sodium and globulin alone. However, nothing is known regarding the relationship between SGR parameters and clinical outcomes. The current study, therefore, sought to validate SGR as a prognostic factor for cancer patients. To the best of our knowledge, this is the first study reporting that high-SGR GC patients, receiving first-line chemotherapy, have better DCR rates than low-SGR counterparts. This affirms the importance of SGR as an independent prognostic factor for PFS and OS in patients with advanced GC. In addition, our results revealed SGR was still a superior prognostic factor, owing to excellent prognosis, based on normal neutrophil counts, serum sodium and globulin level, and without peritoneal metastasis, in patients with advanced GC.

Electrolyte disturbance represents one of the complications for patients with malignancies. Previous studies have implicated sodium reduction in occurrence of this complication, which is associated with poor prognosis in cancer patients 3. In addition, studies have shown that nearly 47% of patients with different malignant diseases exhibit a decrease in serum sodium levels, which is closely related to poor prognosis 6. Functionally, abnormal functioning of several pumps or ion channels across various types of cancer could lead to disease development and progression 18. For example, studies have reported an association between signal transduction of serum and glucocorticoid-induced protein kinase 1 (SGK1) with occurrence and progression of malignancies, including gastric cancer 19. Similarly, a reduction in sodium levels was found to affect maintenance (or enhancement) of SGK1 signaling, indirectly maintaining the tumor-promoting activity of SGK1 20. In addition, several studies have demonstrated that Voltage-gated Na (+) channels (VGSC) implicated in the excitability and action potential conduction were involved in the progression of lung, prostate, breast and gastric cancers 21. In addition, Carrie et al. 22 reported upregulation of VGSC in aggressive tumor cell lines, with its activation found to mediate the invasive potential of cancer cells. Recently, sodium was implicated in immune response processes. For example, Sodium-related receptor potential channels reportedly triggered T cell activation and innate response 23. Previous studies have also shown that sodium impedes differentiation of macrophages into M2 subtypes with immunosuppressive characteristics, while promoting formation of anti-tumoral M1 phenotypic macrophages. Specifically, a decrease in sodium concentration resulted in production of more M2 macrophages and formation of an immunosuppressive microenvironment, which contributed to chemoresistance 24-28. These findings indicate that chemotherapy may generate poor outcomes in GC patients with hyponatremia.

Abundance of inflammatory cells and their products in the tumor microenvironment has been described as the driving factor for formation and progression of tumors 29. Similarly, an increase hepatocyte-derived globulin levels has been reported following inflammatory response 30. Functionally, globulin contains most of the pro-inflammatory proteins, including alpha1-globulin, C-reactive protein (CRP), and complement 3 (C3). Several reports have shown that an abnormal increase in these biomarkers has profound negative effects on cancer patients. For example, Qu et al. 31 reported that higher serum α1-globulin was associated with higher pathological stage and poor tumor status. Consequently, serum α1-globulin was found to be an independent prognostic factor for short recurrence survival and overall survival in non-small cell lung cancer. Similarly, Allin et al. 32 showed that people with high CRP levels, across a general population, had a 1.3-fold higher risk of any type of cancer as well as a 2-fold higher risk of lung cancer. Among cancer patients, those with high CRP levels had 80% risk of premature death, whereas those with elevated CRP levels with invasive breast cancer had a 1.7-fold risk of tumor-related death. In addition, Shimura et al. 33 demonstrated that serum CRP levels, before chemotherapy, might be a potential prognostic factor for metastatic GC, whereas Boire et al. 34 reported upregulation of C3 in leptomeningeal metastatic models, a common fatal condition, and further proved that this was essential for the spread of cancer cells in the leptomeningeal space. The aberrant expression of C3 from cancer cells indicates the relapse of the leptomeninges, with blocking of C3 signal found to effectively inhibit leptomeningeal metastasis. A recent study on GC has shown that C3 deposited in the tumor microenvironment can independently activate the JAK2/STAT3 pathway, and promote tumor progression, indicating its potential as a prognostic factor for patients with gastric cancer 35.

Despite the potential for sodium and globulin alone as prognostic markers for patients with malignancies, these variables are easily influenced by fluid retention and body fluid loss. To circumvent this issue, we evaluated SGR and found it to be a more powerful prognostic factor than sodium and globulin alone owing to higher AUC and lower *p* values in the ROC curve. This possibly reduces the adverse effects of other pathophysiological situations thereby improving its prognostic accuracy.

To our knowledge, this is the first study implicating SGR as a prognostic marker for advanced GC patients. Our findings not only indicate the benefits of first-line chemotherapy, but also the survival of patients. Notably, SGR may still be an effective prognostic predictor for patients with relatively good clinical outcomes. Taken together, these findings are expected to improve prognosis of GC patients and elucidate the actual disease mechanisms. Moreover, our results are expected to guide clinicians during patient identification as well as development of appropriate treatment therapies.

The study had some limitations. Firstly, this was a retrospective study. A randomized controlled approach may be appropriately convincing. Secondly, only sodium and globulin, but not other indicators that contribute to electrolyte disturbance and systemic inflammation, were detected in this study. It is therefore not clear whether these are better prognostic factors. In future, longer follow-up times and detection indexes are needed to supplement these results. Thirdly, although we have described possible mechanisms of action that enable sodium and globulin to be used as prognostic factors in GC cancer patients, biological mechanisms underlying the prognostic role of electrolyte disorders and systemic inflammatory factors remain unknown. Further studies are therefore required on that front.

## Conclusion

SGR, before first-line chemotherapy, is a cheap, universally available, and easily applicable marker that can effectively predict sensitivity to chemotherapy. This factor is also an independent and reliable prognostic factor for PFS and OS in patients with advanced GC.

## Figures and Tables

**Table 1:** Relationship between the pretreatment SGR and clinicopathological features

	Total	Low-SGR	High-SGR	*p*-value
Total (n)	265	157	108	
Age (years, median)	60.0 (52.5-65)	60 (53.0-64.0)	60 (52.0-66.8)	0.435
**Sex (n )**				
Male	175 (66.0%)	106 (67.5%)	69 (63.9%)	0.540
Female	90 (34.0%)	51 (32.5%)	39 (36.1%)	
Body Mass Index (kg/m², median)	21.6 (19.6-23.6)	22.0 (19.8-23.9)	21.4 (19.4-23.2)	0.152
**ECOG (n)**				
0-1	225 (84.9%)	134 (85.4%)	91 (84.3%)	0.807
≥2	40 (15.1%)	23 (14.6%)	17 (15.7%)	
**Histological type (n)**				
Well, Moderately	75 (28.3%)	39 (24.8%)	36 (33.3%)	0.132
Poorly, Mucinous	190 (71.7)	118 (75.2%)	72 (66.7%)	
**The number of organs affected by metastasis (n)**				
0-1	176 (66.4%)	94 (59.9%)	82 (75.9%)	0.007
≥2	89 (33.6%)	63 (40.1%)	26 (24.1%)	
**Peritoneal metastasis (n)**				
YES	79 (29.8%)	61 (38.9%)	18 (16.7%)	< 0.001
NO	186 (70.2%)	96 (61.1%)	90 (83.3%)	
**TNM stage (n)**				
III	60 (22.6%)	30 (19.1%)	30 (27.8%)	0.098
IV	205 (77.4%)	127 (80.9%)	78 (72.2%)	
**Blood parameters and index (median)**				
Albumin (g/L)	40.0 (37.0-42.3)	40.1 (37.0-42.2)	39.9 (36.0-43.0)	0.631
BUN (mmol/L)	5.2 (4.2-6.5)	5.3 (4.2-6.5)	5.2 (4.3-6.8)	0.804
CREA (µmoI/L)	61.5 (50.1-72.2)	61.5 (48.2-73.0)	61.4 (52.7-71.0)	0.656
Potassium (mmol/L)	4.2 (3.9-4.4)	4.2 (3.9-4.4)	4.2 (3.9-4.5)	0.758
Neutrophil counts (×10^9^/L)	3.8 (3.0-5.1)	4.1 (3.3-5.3)	3.5 (2.6-4.5)	< 0.001
Thrombocyte counts (×10^9^/L)	257.0 (196.0-332.5)	266.0 (198.5-341.5)	250 (192.3-313.5)	0.145
Hemoglobin (g/L)	122.0 (105.0-139.0)	123.0 (106.0-140.0)	121.0 (102.0-136.8)	0.161
CEA (ng/mL)	3.8 (1.6-13.0)	4.1 (1.9-11.1)	3.3 (1.2-15.7)	0.396
CA19-9 (U/mL)	20.4 (8.1-122.4)	21.4 (8.6-154.0)	16.0 (7.6-75.1)	0.235
CA72-4 (U/mL)	8.5 (2.4-24.1)	10.6 (3.2-37.8)	5.4 (2.1-20.2)	0.014

SGR: sodium to globulin ratio; ECOG: Eastern Cooperative Oncology Group; TNM: Tumor-Node-Metastasis; CEA: carcinoembryonic antigen; CA19-9: carbohydrate antigen 19-9; CA 72-4: carbohydrate antigen 72-4.

**Table 2:** Correlations between PFS and SGR and other clinicopathological factors

	Univariate analysis			Multivariate analysis		
	Hazard ratio	95% CI	*p*-value	Hazard ratio	95% CI	*p*-value
Age (≥ 60 years)	0.965	0.757-1.231	0.774			
Sex (male)	1.067	0.825-1.379	0.623			
Body Mass Index (<18.5 kg/m²)	0.868	0.607-1.242	0.439			
ECOG (≥ 2)	1.032	0.736-1.446	0.856			
Histological type (Poorly, Mucinous)	1.283	0.980-1.679	0.070	1.214	0.924-1.596	0.164
The number of organs affected by metastasis (≥2)	1.462	1.131-1.890	0.004	1.320	0.984-1.770	0.064
Peritoneal metastasis (NO)	0.519	0.397-0.679	< 0.001	0.701	0.508-0.967	0.031
TNM stage (IV)	1.237	0.925-1.655	0.151	0.892	0.643-1.237	0.494
CEA (> 5 ng/mL)	1.193	0.931-1.529	0.162	1.195	0.914-1.562	0.192
CA19-9 (> 37 U/mL)	1.060	0.826-1.361	0.648			
CA72-4 (> 6 U/mL)	1.420	1.108-1.821	0.006	1.177	0.902-1.535	0.231
Normal neutrophil counts (≤ 6.3×10^9^/L)	0.622	0.430-0.899	0.012	0.673	0.460-0.985	0.042
Hemoglobin (< 115 g/L)	0.881	0.685-1.132	0.322			
SGR > 5.54	0.471	0.365-0.607	< 0.001	0.539	0.411-0.706	< 0.001

SGR: sodium to globulin ratio; ECOG: Eastern Cooperative Oncology Group; TNM: Tumor-Node-Metastasis; CEA: carcinoembryonic antigen; CA19-9: carbohydrate antigen 19-9; CA 72-4: carbohydrate antigen 72-4; CI: confidence interval.

**Table 3:** Correlations between OS and SGR and other clinicopathological factors

	Univariate analysis			Multivariate analysis		
	Hazard ratio	95% CI	*p*-value	Hazard ratio	95% CI	*p*-value
Age (≥ 60 years)	0.788	0.617-1.006	0.056	0.933	0.720-1.210	0.601
Sex (male)	1.063	0.823-1.373	0.640			
Body Mass Index (<18.5 kg/m²)	1.106	0.774-1.580	0.581			
ECOG (≥ 2)	1.096	0.782-1.535	0.595			
Histological type (Poorly, Mucinous)	1.390	1.063-1.819	0.016	1.272	0.969-1.670	0.083
The number of organs affected by metastasis (≥2)	1.395	1.076-1.810	0.012	1.231	0.932-1.627	0.143
Peritoneal metastasis (NO)	0.560	0.428-0.733	<0.001	0.655	0.481-0.894	0.008
TNM stage (IV)	1.177	0.881-1.572	0.270			
CEA (> 5 ng/mL)	1.193	0.932-1.526	0.161	1.285	0.986-1.674	0.063
CA19-9 (> 37 U/mL)	1.134	0.883-1.455	0.324			
CA72-4 (> 6 U/mL)	1.243	0.974-1.587	0.081	0.987	0.755-1.290	0.922
Normal neutrophil counts (≤ 6.3×10^9^/L)	0.681	0.471-0.986	0.042	0.772	0.522-1.139	0.192
Hemoglobin (< 115 g/L)	0.857	0.667-1.101	0.228			
SGR > 5.54	0.485	0.375-0.628	<0.001	0.574	0.437-0.756	<0.001

SGR: sodium to globulin ratio; ECOG: Eastern Cooperative Oncology Group; TNM: Tumor-Node-Metastasis; CEA: carcinoembryonic antigen; CA19-9: carbohydrate antigen 19-9; CA 72-4: carbohydrate antigen 72-4; CI: confidence interval.
